# S100A8/A9 in Inflammation

**DOI:** 10.3389/fimmu.2018.01298

**Published:** 2018-06-11

**Authors:** Siwen Wang, Rui Song, Ziyi Wang, Zhaocheng Jing, Shaoxiong Wang, Jian Ma

**Affiliations:** ^1^Hunan Cancer Hospital, The Affiliated Cancer Hospital of Xiangya School of Medicine, Central South University, Changsha, China; ^2^Xiangya School of Medicine, Cancer Research Institute, Central South University, Changsha, China; ^3^Hunan Key Laboratory of Nonresolving Inflammation and Cancer, Key Laboratory of Carcinogenesis of Ministry of Health, Key Laboratory of Carcinogenesis and Cancer Invasion of Ministry of Education, Changsha, China

**Keywords:** S100A8, S100A9, inflammation, infection, biomarker

## Abstract

S100A8 and S100A9 (also known as MRP8 and MRP14, respectively) are Ca^2+^ binding proteins belonging to the S100 family. They often exist in the form of heterodimer, while homodimer exists very little because of the stability. S100A8/A9 is constitutively expressed in neutrophils and monocytes as a Ca^2+^ sensor, participating in cytoskeleton rearrangement and arachidonic acid metabolism. During inflammation, S100A8/A9 is released actively and exerts a critical role in modulating the inflammatory response by stimulating leukocyte recruitment and inducing cytokine secretion. S100A8/A9 serves as a candidate biomarker for diagnosis and follow-up as well as a predictive indicator of therapeutic responses to inflammation-associated diseases. As blockade of S100A8/A9 activity using small-molecule inhibitors or antibodies improves pathological conditions in murine models, the heterodimer has potential as a therapeutic target. In this review, we provide a comprehensive and detailed overview of the distribution and biological functions of S100A8/A9 and highlight its application as a diagnostic and therapeutic target in inflammation-associated diseases.

Inflammation is a basic defense mechanism in the human body. Various immunocytes and molecules form an enormous regulatory network during inflammation, eliminating endogenous and exogenous pathogenic substances to protect the body. However, imbalance of the network, such as excessive inflammatory reactions and prolonged inflammatory status, may lead to further tissue damage. S100A8 and S100A9 have already been confirmed to play a decisive role in the development of inflammation. They belong to the S100 family, members of which were first extracted as neural proteins from the bovine brain in 1965. These approximately 10,000 Da proteins were named S100 because of their solubility in 100% saturated ammonium sulfate. Human S100A8 and S100A9 consist of 93 and 113 amino acid residues, respectively, and S100A9 has a truncated isoform with 110 amino acids. S100A8 and S100A9 are released by neutrophils and monocytes, and they can form a stable heterodimer or homodimer both *in vitro* and *in vivo*. Both S100A8 and S100A9 have a helix-loop-helix motif with charged amino acid residues, which is a common feature among S100 family members, resulting in their high affinity for divalent ions, such as Ca^2+^ and Zn^2+^. The combination with divalent ions changes the conformation of S100A8/A9, which is a basis for exerting its corresponding functions. Moreover, S100A8/A9 has dual but related functions in intracellular and extracellular microenvironments. In view of its vital role in the physiology of inflammation, S100A8/A9 is a valuable candidate as both a diagnostic biomarker and therapeutic target for inflammation-associated diseases, and its potential in clinical applications is worthy of further exploration.

## Expression and Distribution of S100A8/A9

S100A8 and S100A9 are mainly derived from immunocytes, such as neutrophils and macrophages, which contain abundant S100A8/A9 and participate in inflammatory process. S100A8 and S100A9 proteins comprise approximately 45% of the cytoplasmic proteins in neutrophils. Under physiological conditions, there is sufficient storage of S100A8/A9 in neutrophils and myeloid-derived dendritic cells, while low levels of S100A8/A9 are constitutively expressed in monocytes. S100A8/A9 is intensely upregulated during trauma, infection, heat, stress, and many other inflammatory processes.

### Infection-Induced Inflammation

Infection-induced inflammation is one of the main resources for S100A8/A9 secretion. After being infected with bacteria, neutrophils, macrophages, and monocytes intensely express and secrete S100A8/A9 to modulate inflammatory processes with the induction of inflammatory cytokines, reactive oxygen species (ROS), and nitric oxide (NO). S100A8 and S100A9 also have antibacterial potential *via* their ability to bind Zn^2+^.

The accumulation of S100A8 and S100A9, which are mainly expressed by mononuclear cells in red pulp, has been observed in mice infected with plasmodium (1). In HIV-1 infected patients, serous S100A8/A9 levels are upregulated and correlated with disease progression and low CD4^+^ T cell counts (2). Influenza A virus (IAV) activated toll-like receptors (TLRs) *via* pathogen-associated molecular patterns and damage-associated molecular patterns, where S100A9 acted as a pro-inflammatory factor. During IAV infection, S100A9 is increasingly released *via* DDX21–TRIF signaling from undamaged macrophages, resulting in an exaggerated inflammatory response and cell death (3). Lipopolysaccharide (LPS) activates the caspase-4/5 inflammasome to promote S100A8 secretion from macrophages. A marked increase in the S100A8/A9 level, which is correlated with the duration of fever before admission in acute phase plasma and feces, was observed in typhoid fever patients (4). S100A9 expression is significantly upregulated in the early stage of *Klebsiella pneumoniae* infection-induced sepsis. In septicemia-induced septic shock, the expression of S100A9 is continuously increased until the patient’s death (5). Deficiency of S100A8/A9 in mice could promote the progression of pneumonia caused by *Staphylococcus aureus* infection (6). There are other types of cells that can release S100A8 and S100A9 upon infection; for example, during hidradenitis suppurativa infection, keratinocytes are one of the most important sources of S100 proteins (7).

The early expression of S100 proteins during infection-induced inflammation suggests that S100A8 and S100A9 participate in innate immunity and mediate the inflammatory response. By triggering TLR4- or RAGE-mediated multiple inflammatory pathways, S100A8/A9 plays an important role in protecting the body from pathogenic infection (8). S100A8/A9 also participates in cytosol tubulin polymerization and cytoskeleton rearrangement, which are essential prerequisites for cell migration and may somehow explain why S100A8/A9 can recruit neutrophils during inflammation.

The expression and secretion of S100A8/9 during infection-induced inflammation are restricted by a negative feedback regulatory mechanism (9). Excessive expression of S100A8/A9 magnifies the inflammatory response and accelerates neutrophils and macrophages to release more cytokines, which induces a vicious cycle and aggravates the disorder. During an infection with Gram-negative bacteria, as a ligand for TLR4, S100A8 is strongly induced in endotoxic shock. High levels of S100A8 and S100A9 activate RAGE signaling and result in inflammatory damage in septic shock patients (10). Although the excessive expression of S100 proteins reveals a strong connection with exacerbation of disease, none of S100A9 is observed increasing in patients with unstable chronic obstructive pulmonary disease (COPD) resulted from infection. The decreasing of S100A9 might indicate the insufficient immunity, which explains the exacerbation induced by infection. However, the severe COPD patients induced by other factors have a high expression of S100A9, which implies the uncontrolled immune reaction. Hence, the proper levels of S100 proteins may contribute to both defense capabilities and immunity homeostasis (11).

### Metabolic Inflammation

In metabolic inflammatory diseases, such as gout, diabetes, and obesity, S100A8/A9 is secreted and distributed in a disease-specific manner, and elevated levels of S100A8/A9 have been detected in sera and inflammatory sites. In gout patients, neutrophils migrate to gout-affected joints and secrete S100A8/A9, which accelerates inflammation (12–14). S100A8/A9 expression is significantly increased in synovia, tophi, and sera of gout patients and is correlated with disease progression. Monosodium urate (MSU), or uric acid, is the etiological agent of gout, an acute inflammatory condition. MSU crystals promote neutrophils and macrophages that express and secrete S100A8/A9, and these S100 proteins enhance MSU-induced activation of the NLRP3 inflammasome in macrophages and neutrophils, which release IL-1β and mediate gout pain. S100A9 was intensely induced in omental adipose tissue in patients with gestational diabetes (15). S100A8/A9 not only extends the damage but also participates in inflammation maintenance. Obesity is a form of metabolic inflammation because it is hard to remove excess fat from the body. Fat-derived S100A8/A9 stimulates the TLR4–MyD88 cascade to enhance the expression of IL-1β mRNA in macrophages, provoking myelopoiesis (16). Higher S100A9 expression in epicardial stromal cells is associated with lower adipocyte sizes in patients with cardiovascular diseases (CVDs). Moreover, smaller epicardial adipocytes produce higher oxidative stress than subcutaneous adipocytes (17). Both cell types and metabolites influence the S100 protein levels. Expression of S100 is promoted in type I diabetes patients, and high levels of expression have been observed in retinal vascular endotheliocytes, white blood cells, fibroblasts, and vitreous of patients with proliferative diabetic retinopathy (PDR) (18–20). S100A9 is highly expressed in glial cells and promotes amyloidosis and amyloid-β (Aβ) aggregation. There is positive feedback between S100A8 and Aβ, as upregulated levels of S100A8 promotes the expression of Aβ2 by interfering with amyloid precursor protein (APP) metabolism, and Aβ contributes to S100A8 translation. At the same time, it is also observed a striking decrease in the expression of S100A8 and S100A9 in CD11c^+^ cells which surround the amyloid plaques and might play a beneficial immune-modulatory role in Alzheimer’s disease (AD). These changes suggest the disorder in the expression of S100A8 and S100A9 among different cells might be critical to explain how the plaques form (21). In addition to its aggressive functions in metabolic inflammation, in some cases, S100A8/A9 plays a defensive role. In diabetic foot ulcer patients, S100 proteins in wound exudates resist bacterial infection. In AD, S100A9 secretion from macrophages is significantly inhibited by Aβ1-42 monomers, which leads to the loss of monocyte function (21–23).

As mentioned earlier, the locations at which S100A8/A9 is elevated vary with the disease. S100A8/A9 is increased in synovial fluid (SF), serum, and tophi of gout patients, while it is increased in the vitreous of PDR patients, which underlines the importance of appropriate specimen selection for the application of S100 proteins as biomarkers.

### Inflammation Caused by Immune System Dysfunction

Dysregulated and excessive immune responses result in autoimmune diseases and hypersensitivity reactions, such as inflammation. Upregulation of S100A8/A9 occurs in multiple immune system dysfunction diseases.

In psoriatic arthritis patients, S100A8/A9 is intensely expressed in the synovial sublining layer, suggesting the importance of S100A8/A9 in mediating leukocyte migration across the endothelium. S100A8/A9 is upregulated not only in the serum and SF but also in psoriatic arthritis plaque. In rheumatoid arthritis (RA), S100A8 is mostly released from activated macrophages, and its expression level is correlated with traditional parameters, such as C-reactive protein (CRP), erythrocyte sedimentation rate, and rheumatoid factors, which implies that the amount of S100A8 may be a good parameter for evaluation. In psoriasis patients, S100A8 is mainly derived from keratinocytes and infiltrating mononuclear cells, and S100A9 is derived from neutrophils (24–26). In systemic lupus erythematosus (SLE) patients, the serum levels of S100A8/A9 released from polymorphonuclear (PMN) cells are elevated and are particularly increased in patients with anti-dsDNA antibodies and glomerulonephritis. Serum S100A8/A9 levels may be used to monitor the disease activity, as higher levels of S100A8/A9 have been detected in patients with active SLE (27, 28). In addition, high levels of S100A8/A9 have been observed in type I hypersensitivity reactions. In human neutrophils, S100A8/A9 presents its capability of binding arachidonic acid (AA), which are significant mediators of asthma. S100A9 is overexpressed in asthma and may be a potential regulator that reveals the role of neutrophils in amplifying airway inflammatory responses (29). In food allergies, S100A8/A9 in the feces and TLR4, NF-κB, IL-1β, and IL-6 in the liver and jejunum are elevated, which indicates that S100A8/A9 regulates the balance of Th1/Th2 and amplifies the allergic cascade (30).

S100A8/A9 contributes to multiple immune-associated diseases *via* various pathways. S100A8/A9, a TLR4 ligand, is abundant, and its level has a marked correlation with IL-6 and IL-7 levels in SF of RA patients. As the most abundant protein in RA SF, S100A8 has a crucial role in promoting IL-6 expression in fibroblast-like synoviocytes *via* TLR4/PI3K/NF-κB and MAPK signaling (31, 32). S100A8/A9 participates in the progression of psoriasis. The high expression of S100A8/A9 in psoriasis epidermis induces C3/CFB complement activation, which subsequently leads to uncontrolled immunocyte activation, angiogenesis and keratinocyte hyperproliferation. Barrier-to-autointegration factor 1 (BANF1) is an essential component of nuclear lamina, and strong nuclear-dominant immunostaining of BANF1 was seen in the epidermal keratinocytes of psoriatic lesions. Activation of BANF1 suppresses S100A9 expression and inactivates c-Jun, resulting in the suppression of cutaneous inflammation (33). In fact, the level of S100A8/A9 is related to skin barrier dysfunction; in specific dermatitis, upregulated S100A8/A9 expression exacerbates immune-induced damage, while the condition is improved with defects in MyD88. In addition, IL-6–STAT–SOCS3 is a negative feedback axis mediating epidermal repair and inflammatory homeostasis. With SOCS3 deficiency, IL-6 strongly induces S100A8/A9 expression, resulting in excessive epidermal proliferation and angiogenesis (24–26).

### Inflammation Caused by Degenerative Diseases

Degenerative diseases are chronic and progressive inflammation-related disorders that have multiple pathophysiological factors, especially age. A shift in the abundance of S100A8/A9 is a robust feature of aging in mammalian tissues, involving a range of cell types, including the central nervous system, which suggests that S100A8/A9 may be involved in age-related inflammation (34). In patients with osteoarthritis (OA), S100A8/A9 mainly exists in GM-CSF-derived macrophages of the synovial membrane. Stimulated by IL-1, chondrocytes express and release large amounts of S100A8 and S100A9, and extracellular S100A8 then stimulates the synovial membrane to generate pro-inflammatory cytokines, such as IL-1β, IL-6, IL-8, TNF-α, and MMPs, which facilitates an inflammatory environment and promotes cartilage degradation (35–37). Influx of ox-LDL in the joint promotes monocytes and neutrophils to release S100A8/A9, resulting in joint injury *via* the TGF-β signaling pathway, and OA should thus be associated with cholesterol (38). In an experimental OA mouse model, high-cholesterol food induced ApoE^−/−^ mice to highly express S100A8/A9, leading to synovial activation and cartilage degradation.

Immunocytes (macrophages, neutrophils, etc.) and cells in local lesions are the main sources of S100A8/A9, which are consistently elevated in degenerative diseases related to inflammation. SPI/PU.1 is reportedly an S100A9 transcription driver, and SATB1 is a transcription inhibitor. In inflammation caused by degeneration, SPI/PU.1 promotes S100A9 sustained expression *via* a positive feedback mechanism, inducing inflammation by activation of RAGE, NF-κB, and other signaling pathways (34). Therefore, the imbalance of S100A8/A9 expression may be one of the most significant mechanisms of degeneration related to inflammation.

## Biological Functions of S100A8/A9

The unique structures of S100A8/A9 endow them with the potential to play multiple roles, which depend on concentrations, posttranscriptional modifications, and oligomeric forms as well as proximal microenvironments. Intracellular S100A8/A9 complexes participate in cytoskeleton modulation, AA metabolism, and protection against pathogens. In addition to the ability to stimulate leukocyte recruitment and cytokine secretion, extracellular S100A8/A9 also exhibits anti-inflammatory properties under specific conditions, suggesting that these proteins contribute to homeostasis during inflammation. Moreover, S100A8/A9 exerts antimicrobial function and participates in the modulation of cell proliferation, differentiation, and apoptosis. This section will provide a comprehensive overview on the intracellular and extracellular functions of S100A8/A9.

### Intracellular Activities of S100A8/A9

Intracellular S100A8/A9 is suggested to be a Ca^2+^ sensor; binding to Ca^2+^ changes its conformation and modulates Ca^2+^-dependent signaling. Moreover, S100A8/A9 exerts both regulatory and protective functions in the cytosol.

#### Cytoskeleton Modulation

S100A8 and S100A9 mediate the rapid rearrangement of cytoskeleton, which is a prerequisite for successful cell migration, phagocytosis, and exocytosis. Translocation of the cytosolic S100A8/A9 complex to the plasma membrane is Ca^2+^ dependent and so is the interaction between S100A8/A9 and cytoskeletal proteins, including tubulins, microfilaments, and keratin intermediate filaments in activated phagocytes and epithelial cells (39–42). S100A8/A9 complex plays a significant role in microtubule polymerization and stabilization in resting phagocytes, while S100A8 directly binds to tubulin and S100A9 functions as a regulatory subuint. The phosphorylation of S100A9 through Ca^2+^ and p38 MAPK signaling during inflammation reverses microtubule formation and leads to rearrangement cytoskeleton, resulting in effective leukocyte migration (41). Leukocyte migration in *S100A9*-knockout mice is deficient, and there is a reduction in polymerized microtubulin in *S100A9^−/−^* neutrophils, in which impaired activation of small GTPases Cdc42 and Rac is detected, supporting an indispensable role for intracellular S100A8/A9 complex in cytoskeletal modulation (41). Remarkably, Ca^2+^-induced (S100A8/A9)_2_ heterotetramer, but not heterodimer, seems to play a crucial role in stabilizing microtubule network as disturbed tetramerization is associated with impaired microtubule formation (43). It is assumed that the tetramer of S100A8 and S100A9, in which there are more binding sites for tubulin, is superior to dimer in bundling and crosslinking microtubules.

#### Transfer of Polyunsaturated Fatty Acids and Activation of Nicotinamide Adenine Dinucleotide Phosphate (NADPH) Oxidase

The intracellular S100A8/A9 complex is implicated in respiratory burst. In the presence of Ca^2+^, S100A8/A9 binds to polyunsaturated fatty acids in the cytosol, such as AA and α- and γ-linolenic acid, in a saturable and reversible manner (44, 45). The binding of Ca^2+^ to each EF hand within S100A8/A9 is a prerequisite for AA binding, whereas Zn^2+^ or Cu^2+^ binding induces conformational changes different from that of Ca^2+^ binding, affecting the formation of the AA binding pocket in the protein complex and abrogating its capacity to transfer AA (46). The delivery of AA to the membrane-bound gp91phox subunit boosts the activation of NADPH oxidase, generating ROS in phagocytes (47). The activation may be further enhanced by S100A8/A9 acting as a scaffold between NADPH oxidase and AA to facilitate their interaction (48, 49). Furthermore, the binding capacity of S100A8/A9 facilitates AA transcellular transport during inflammation. The S100A8/A9–AA complex may be internalized by infiltrated cells at inflammatory foci for the synthesis of inflammatory mediators, such as leukotriene B4, which can trigger leukocyte degranulation as well as cellular damage to vascular endothelium, contributing to the initiation and regulation of inflammatory responses (50).

#### Resistance to Pathogens

One-third of bacteria bind to S100A8/A9-expressing cells, in which only one-tenth of intracellular microorganisms exist, demonstrating that S100A8/A9 in the cytosol attenuates bacterial adherence and invasion (51). Similarly, transfection with S100A8/A9 expression vectors into epithelial cells augments cellular resistance to invasion by *Listeria* and *Salmonella* (52). For neighboring keratinocytes, S100A8/A9-dependent resistance to *Listeria* could be provoked by IL-1α from infected epithelial cells in a paracrine manner (53). However, by inducing the translocation of intracellular S100A8/A9 to microtubules, *Listeria* appears to weaken antimicrobial activity of S100A8/A9, leading to an increased amount of bacteria in keratinocytes (51). Notably, Ca^2+^-binding loops I and II in S100A9 are essential for keratinocyte resistance to bacterial invasion and intermolecular stability, as mutations in these loops cause a complete loss of the intracellular antibacterial activity of the S100A8/A9 complex (54).

### Extracellular Activities of S100A8/A9

#### Leukocyte Recruitment

Mouse S100A8 is the first S100 family member found to have potent chemokine-like activity to murine phagocytes both *in vitro* and *in vivo* and was once termed chemotactic protein 10 kDa to reflect its function. Injection of mS100A8 stimulates the early recruitment of neutrophils followed by monocytes over 24 h, with kinetics similar to delayed-type hypersensitivity responses elicited by antigen injection into a sensitized host (55). In contrast to classical chemokines, mS100A8 leads to actin polymerization and profound shape changes in phagocytes at picomolar levels *via* a pertussis toxin-sensitive, G-protein coupled pathway with no influence on the intracellular Ca^2+^ level or integrin or L-selectin expression (47).

The treatment of neutrophils with S100A9 enhances transendothelial migration, while blockage with anti-S100A9 antibodies diminishes leukocyte infiltration in the joints of a murine arthritis model. Blockage of RAGE but not TLR4 inhibits the S100A9-mediated recruitment of macrophages and leukocytes *in vitro* (56). In response to chemokines, *mS100A9^−/−^* neutrophils have reduced Ca^2+^ influx and migration, indicating that abnormal cytoskeletal dynamics may be responsible for altered chemotaxis (57). Treating *mS100A9^−/−^* mice with G-CSF reverses impaired neutrophil recruitment into infected lungs in response to *pneumococcal* infection, suggesting that mS100A9 regulates chemotaxis by driving the production of G-CSF (58). The chemotactic activity of S100A8 may be modulated by oxidation, as S100A8 is inactivated by hypochlorous acid treatment with the formation of intermolecular sulfonamide-linked complexes. However, S100A9 expressed by epithelia in healthy mucosal tissue exerts a chemo-repulsive effect on peripheral leukocytes, which is abrogated by the oxidation of methionine 63 and 83, suggesting a role of S100A9 as a molecular switch of inflammation in oxidative conditions (59). Conversely, human S100A8 exerts contentious leukocyte chemotactic activity, as there is only a 21% identity of amino acids between hS100A8 and mS100A8 within the hinge region and neighboring α-helix, which is responsible for chemotaxis (41, 60).

In addition to chemotactic function, mS100A8 as well as hS100A8 and hS100A9 stimulate leukocyte migration by upregulating the expression of adhesion molecules and enhancing leukocyte–endothelial cell interaction. Moreover, these proteins alter the intercellular contacts between endothelial cells and increase vascular permeability, facilitating leukocyte extravasation. S100A8 and S100A9 induce neutrophil adhesion to fibrinogen *in vitro via* upregulating Mac-1 (a heterodimer of CD11b and CD18) expression and increasing L-selectin shedding, which is also associated with an elevated intracellular Ca^2+^ level (61), while *S100A9^−/−^* neutrophils exhibit impaired Mac-1 expression and a reduced capacity to migrate through endothelial cells (62). Cellular interaction during neutrophil rolling triggers S100A8/A9 secretion. The release of S100A8/A9 induces VCAM-1 and ICAM-1 expression in endothelial cells and augments the capacity of leukocyte Mac-1 to bind endothelial ICAM-1 in a TLR4-mediated, Rap1-GTPase-dependent pathway, resulting in reduced rolling velocity and fastened adhesion for transendothelial migration (63, 64).

In bleomycin-induced lung injury, mS100A8 in the inflammatory microenvironment contributes to leukocyte recruitment in the early stage of lung damage (65). Similarly, acute migration of neutrophils into the vagina is mediated by mS100A8 and mS100A9 released from vaginal epithelial cells in a *Candida* infection mouse model (66). In mice undergoing tibial fracture surgery, upregulated mS100A8 induces neutrophil infiltration and microglia activation in the hippocampus, promoting the occurrence and development of neuroinflammation and postoperative cognitive dysfunction *via* TLR4/MyD88 signaling (67). For type 1 diabetic patients, increased S100A8/A9 upregulates CD11b expression of monocytes and induces adhesion to fibrinogen, facilitating the accumulation of monocyte-derived cells in pancreatic islets (68). *S100A9^−/−^* mice are less sensitive to LPS stimulation and more resistant to LPS-induced septic shock (69). Pretreatment with anti-mS100A8 and anti-mS100A9 antibodies reduces migration of neutrophils and macrophages to the alveoli by 70 and 80%, respectively, in mice with streptococcal pneumonia infection, without impairing leukocyte blood count or neutrophil sequestration in the lung vasculature (70). Thus, blocking S100A8 and S100A9 might represent a novel modality for inhibition of leukocyte recruitment in treating inflammation-associated diseases.

#### Cytokines

**Figure 1:**
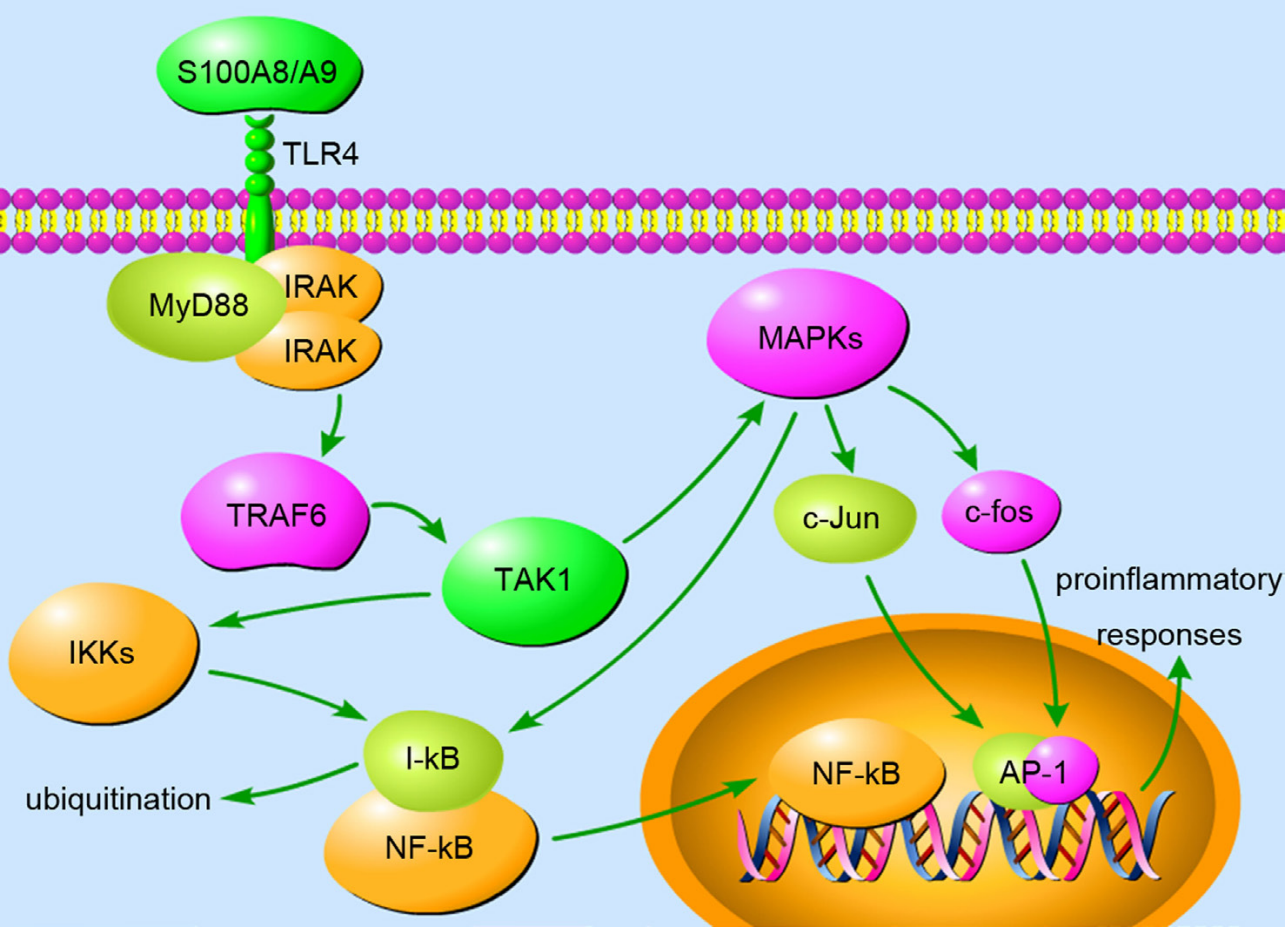
The binding of S100A8/A9 to toll-like receptor (TLR) 4 triggers the MyD88-dependent pathway, appearing to play a vital role in inflammation. MyD88 recruits and activates IRAKs and TRAF6, which activates TAK1 *via* a TAB-mediated method. The activation of TAK1 facilitates the phosphorylation and activation of IKK and MAPK. Activated IKK phosphorylates and ubiquitinates I-κB, resulting in the liberation of NF-κB from inhibition. NF-κB translocates to the nucleus and induces the expression of pro-inflammatory cytokines. MAPKs, including p38, JNK, and ERK, have been demonstrated to activate c-Jun and c-fos, which translocate to the nucleus and form heterodimer AP-1, another transcription factor. Moreover, MAPK pathways are also involved in S100A8/A9-induced NF-κB activation during inflammation. The upregulated expression of pro-inflammatory genes amplifies the inflammatory response and severely destructs tissues in inflammation-associated diseases.

The release of S100A8 and S100A9 can induce the secretion of multiple cytokines in inflammatory cells to sustain and exacerbate inflammation. As endogenous ligands of TLR4, S100A8, and S100A9 enhance MSU crystal-induced IL-1β secretion in phagocytes, both *in vitro* and *in vivo* (13). During septic shock, S100A8 induces translocation of MyD88, hyperphosphorylation of IRAK-1, and activation of NF-κB, resulting in elevated expression of TNF-α in phagocytes (69). Increased levels of S100A8/A9 in gingival crevicular fluids of periodontitis patients induce IL-6 production of gingival fibroblasts *via* TLR4 signaling involving MAPK and NF-κB (71). In BV-2 microglia, S100A8/A9 stimulates the production of TNF-α and IL-6 through ERK/NF-κB and JNK/NF-κB signaling (72). Blockage of S100A8/A9 or downstream signaling reduces pro-inflammatory cytokine secretion and ameliorates excessive inflammation. However, S100A9 is not a direct activator of cytokine expression in human neutrophils, but it potentiates IL-8 secretion induced by other neutrophil activators including fMLP and GM-CSF, following NF-κB, CREB-1, and STAT3/STAT5 activation (73). In addition, treating human monocytes with S100A9 increases the secretion of IL-1β, IL-6, and TNF-α in a process intimately linked to ROS generation (74). In a murine arthritis model, treatment with anti-S100A9 antibodies diminishes pro-inflammatory cytokine levels both in joints and in serum and preserves bone/collagen integrity (75). In response to TLR4 stimulation, S100A9-deficient neutrophils exhibited impaired production of cytokines (75). S100A9 and S100A8/A9 significantly upregulated IL-6 and IL-8 expression in human gingival fibroblasts *via* the S100A9 subunit (76). (Figure 1 demonstrates the S100A8/A9-induced inflammation signaling pathway involved in TLR4, MyD88, and MAPKs.) The above observation clearly revealed the significance of S100A8 and S100A9 in promoting cytokine secretion under inflammatory conditions.

#### Anti-Inflammatory Function

Although much attention has been paid to the pro-inflammatory functions of S100A8/A9, the complex also exhibits anti-inflammatory properties under specific conditions to avoid tissue damage caused by overwhelming inflammation. S100A8, S100A9, and S100A8/A9 have been demonstrated to modulate production of pro-inflammatory mediators, including cytokines, chemokines, ROS, and NO. S100A8, *in vitro*, reduces mast cell degranulation and secretion of IL-4, IL-6, and GM-CSF in response to IgE-crosslinking by inhibiting intracellular ROS production. In the lungs of acute asthma mice, S100A8 suppresses mast cell degranulation, eosinophil chemoattractant production, and eosinophil infiltration (77). Prestimulation of both murine and human monocytes with S100A8 attenuates IL-6 and TNF-α production in response to LPS and bacteria *via* downregulation of phosphorylated p38, thus protecting the host against lethal sepsis (9). Moreover, non-covalent and high-affinity binding of S100A8/A9 with pro-inflammatory IL-1β, IL-6, and TNF-α suggests the capacity of S100A8/A9 to trap cytokines (78, 79). S100A8/A9 inhibits oxidative metabolism of PMNs *in vitro* and scavenges released ROS, ameliorating oxidative damage in lungs and livers of LPS-treated mice (80). S100A8 promotes anti-inflammatory IL-10 expression in airway epithelial cells, resulting in impaired LPS-induced neutrophil infiltration and reduced pro-inflammatory cytokine induction (81).

S100A8 also negatively regulates leukocyte adhesion and transmigration through reducing p38 MAPK phosphorylation (41). S100A9 inhibits B7 expression to reduce antigen presentation by dendritic cells and subsequent T cell priming, preventing hyperactivation of the adaptive immune system (82). In addition, S100A8/A9 exerts regulatory activity in inflammation through its growth-inhibiting and apoptosis-inducing potentials. In the resolution phase of inflammation, apoptotic cells are cleared through phagocytosis, and phagocytosis in turn generates inhibitory signals for the pro-inflammatory activation of macrophages. The phagocytic activity of macrophages is restored when cultured in conditioned medium of neutrophils previously depleted of S100A9, indicating that S100A9 is a key player in suppression of pro-inflammatory activation of macrophages (83). In conclusion, S100A8, S100A9, and S100A8/A9 participate in modulation and restoration of homeostasis during inflammation, but their excessive expression and secretion may lead to an imbalance of inflammatory processes.

#### Antimicrobial Function

Once released into the extracellular space from infiltrating phagocytes or after cell necrosis, the S100A8/A9 complex exhibits broad-spectrum antimicrobial activity against numerous microorganisms. This activity is mediated by the ability of S100A8/A9 to bind and control the levels of essential trace metals such as Zn^2+^ and Mn^2+^, which are required for bacterial growth (84, 85). Both binding sites for Zn^2+^ and Mn^2+^ in S100A8/A9 are necessary for its antimicrobial function, as recombinant the S100A8/A9 complex with mutations in either site has impaired antimicrobial function (86). Since local levels of Zn^2+^ and Mn^2+^ could modulate the affinity between S100A8/A9 and its targets including bacteria, the antimicrobial potential may be diverse in various pathological states.

Purified hS100A8/A9 has been described to inhibit the growth of multiple species *in vitro*, including *Escherichia coli, Candida albicans, S. aureus, K. pneumoniae, Salmonella typhimurium*, and *Listeria monocytogenes* (51, 87, 88). S100A8/A9 in mucosal fluids, airway secretions, gingival crevicular fluid, and tissue abscesses contribute to the limitation of commensal microorganism growth and prevention against the intrusion of pathogens (89).

In addition to its metal chelating property, S100A9 has been found to enhance the efficiency of human neutrophil phagocytosis in a Syk-, Erk1/2-, and PI3K/Akt-dependent manner, thereby augmenting its antimicrobial activity toward *K. pneumoniae* as well as *E. coli* (90). Lack of S100A8/A9 in mice leads to a significant increase in the bacterial burden in blood, liver, and spleen (91). Hence, S100A8/A9 inhibits the growth of pathogens at infectious sites during the initial phase of infection, allowing time for the recruitment of phagocytes, and then, S100A9 enhances the phagocytic activity of infiltrating leukocytes, accelerating the clearance of pathogens.

#### Alzheimer’s Disease

Alzheimer’s disease, in which neuroinflammation plays a fundamental role, is characterized by extracellular amyloid plaques and intraneuronal neurofibrillary tangles (92). Significant upregulation of S100A8 and S100A9 is detected within amyloid plaques and neighboring activated microglia in the brains of AD mice as well as AD patients (93, 94). S100A8/A9 induces extensive activation of microglia and expression of multiple inflammatory factors including TNF-α and IFN-γ, which induce the transcriptional activity of BACE1 (β-secretase 1) and BACE2 promoters, resulting in increased production of β-CTF (β-secretase-cleaved C-terminal fragment of APP, the direct precursor of Aβ) and subsequently, increased Aβ generation (95). The interaction between Aβ and S100A9, which is triggered by Aβ binding to the S100A9 hinge region, accelerates the formation of fibrillar amyloid structures and reduces S100A9-mediated cytotoxicity (96). Moreover, aggregation of S100A8 is seen before Aβ deposition in mouse AD models, suggesting the existence of positive feedback between S100A8 and Aβ expression (94). In Tg2576 mice, there is improved memory function and neuropathology, accompanied by reduced Aβ and amyloid plaque burden after S100A9 was knocked out (77). In the amyloid precursor protein/presenilin1 (APP/PS1) mouse model, loss of S100A9 ameliorates amyloid burden by increasing microglial phagocytosis of fibrillar amyloid and modulating APP processing (95). Analogously, antibodies of S100A9 reverse impaired passive avoidance learning in C57BL/6 mice caused by chronic intranasal administration (97). Thus, S100A9, together with S100A8 to a lesser extent, serves as a strong link between inflammatory cascades and amyloid plaques and has a considerable therapeutic potential for AD patients.

#### Modulation of Cellular Proliferation, Differentiation, and Apoptosis

S100A8/A9 exerts proliferative activity at lower protein concentrations. S100A8/A9 at 10 µg/ml induces significant growth-promoting activity in MCF-7, MDA-MB231, and SHEP breast cancer cell lines, whereas the S100A8/A9 protein at higher concentrations does not enhance cellular proliferation (98). At 100 ng/ml, S100A8/A9 stimulates the growth of NHK cells, but 10 µg/ml S100A8/A9 suppresses it (26). S100A8/A9 at low concentrations promotes tumor cell growth through RAGE signaling and activation of NF-κB (98). In the colitis-associated cancer mouse model, colonic chitinase 3-like 1 (CHI3 L1) can bind to RAGE, and thus disrupt the S100A9-associated expression positive feedback loop during early immune activation, creating a S100A9 low colonic environment, especially in the later phase of colitis. Low concentrations of S100A9 promote cell proliferation/survival of both normal intestinal epithelial cells and tumor cells in this mouse model (99). S100A8/A9 treatment rapidly induces phosphorylation of p38 and p44/42 MAPKs in MCF-7 and MDA-MB231 cells with an increase in NF-κB activity, although p38 MAPK inhibitor and p44/42 MAPK inhibitor can reverse the proliferative effect of S100A8/A9 on these cell lines (98). Therefore, S100A8/A9 binds to RAGE and subsequently induces phosphorylation of p38- and p44/42 MAPK as well as activation of NF-κB.

S100A8 and S100A9 are regulators of myeloid differentiation in leukemia (100). S100A9 induces acute myeloid leukemia (AML) cell differentiation through TLR4–MAPK/ERK–JNK signaling, whereas S100A8 prevents differentiation induced by S100A9 activity and maintains the AML immature phenotype. Recombinant S100A9 significantly diminishes symptoms and prolongs survival of AML mice. Interestingly, anti-S100A8 antibody treatment had effects similar to those of S100A9 therapy *in vivo*, suggesting that high ratios of S100A9 to S100A8 are required to induce AML differentiation.

**Figure 2:**
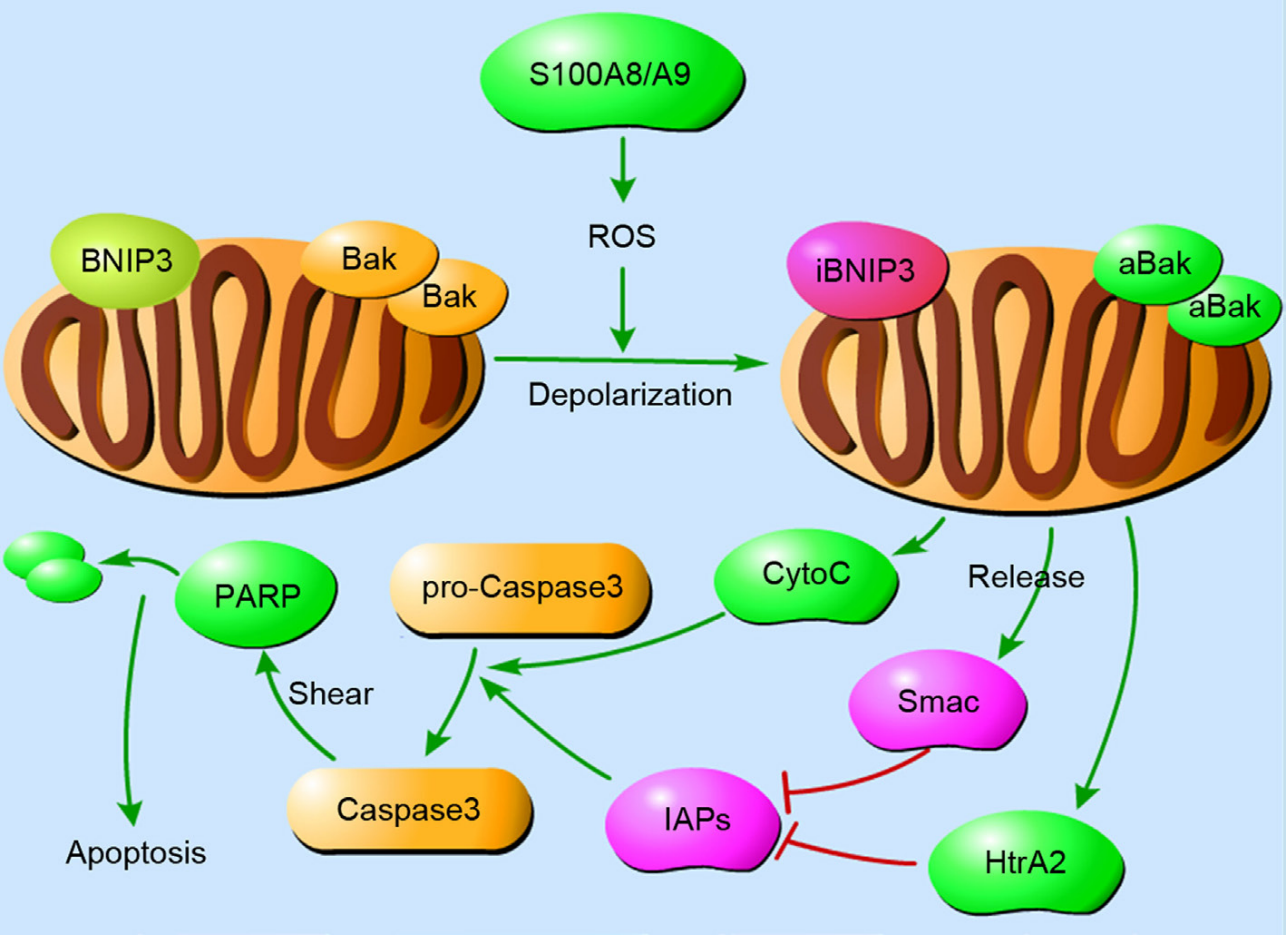
The mitochondrial pathways of S100A8/A9-induced apoptosis. Cellular apoptosis is a cascade that involves a rapid drop in mitochondrial membrane potential. S100A8/A9 increases reactive oxygen species (ROS) production, resulting in the depolarization of mitochondrial membranes and subsequent inhibition of BNIP3, an atypical pro-apoptotic Bcl2-family member, and activation of Bak. After the translocation of Bak and BNIP3 in mitochondria, cytochrome C, Smac, and HtrA2 are concomitantly released from mitochondria into the cytoplasm. They activate caspase-3 in a direct way or in an IAPs-dependent manner to induce apoptosis. Abbreviations: iBNIP3, inhibited BNIP3; aBak, activated Bak.

S100A8/A9 has the capacity to induce apoptosis in various cells under inflammatory conditions. It has been described previously that S100A8/A9 suppresses the growth of yeast and fungi, and the minimum effective concentration is between 10 and 20 mg/ml (87, 101). This growth-inhibitory activity is also found in mammalian cells such as macrophages, bone marrow cells, lymphocytes, and fibroblasts (102–105). The ability to induce apoptosis is regulated by the concentration of S100A8/A9 and other proteins. For example, a high concentration (10 µg/ml) of S100A8/A9 induces apoptosis in NHKs but exerts cell growth at a low concentration (10 ng/ml) (26). Synthesis of apoptosis cascade proteins and the release of ROS may be essential elements in the death-inducing function of S100A8/A9. (Figure 2 demonstrates the S100A8/A9-induced mitochondrial apoptosis pathways.) In addition, Zn^2+^ exclusion from the target is one of the most significant mechanisms employed by S100A8/A9 because Zn^2+^ effectively inhibits apoptosis (106). The other mechanism may be the binding of S100A8/A9 to the target cell surface in a ligand–receptor manner (107, 108).

These modulatory capacities of S100A8, S100A9, and S100A8/A9 suggest that they play a regulatory role in inflammation *via* their effect on the survival state of various cells. Specifically, their presence at local inflammatory sites might cause either tissue proliferation/repair at low concentrations or deleterious effects on the inflammatory tissue at high concentrations.

## S100A8/A9 as a Biomarker

S100A8/A9 could be used as a biomarker in many inflammatory diseases, such as juvenile RA (109), inflammatory arthritis disease (110), skin stresses (111), transplantation (112), inflammatory bowel disease (IBD) (113), islet inflammatory response, severe forms of glomerulonephritis (68), cystic fibrosis (114), periodontitis, autoimmune synovitis (115), inflammation of the uterine cervix (116), peritonitis (117), microcirculatory defects in diabetic nephropathy (118), infections (119, 120), CVDs (121, 122), and autoimmune diseases such as juvenile dermatomyositis (123).

The serum S100A8/A9 level of patients with systemic inflammatory response syndrome and sepsis showed a significant increase compared with healthy controls; S100A8/A9 can be an independent predictor of 28-day mortality and a promising biomarker in early diagnosis, evaluation of prognosis and risk stratification (124). S100A8 and S100A12 in amniotic fluid from pregnant women were found to be the strongest predictor of increased early-onset sepsis incidence in neonates of pregnant women (125, 126).

A large number of inflammatory molecules are found in diabetes, including S100A8/A9 (19). S100A8/A9 has been used as a biomarker of diabetes mellitus (68), and high levels of this protein complex may be associated with atherosclerosis in diabetic patients (127). In addition, the level of S100A8/A9 indicates the inflammatory environment of type 2 diabetic nephropathy and varying degrees of microvascular lesions in the glomeruli and retina, becoming a potential new biomarker for microcirculation defects in diabetic nephropathy (118). Moreover, compared with non-obese healthy individuals, the level of plasma S100A8/A9 in obese individuals is higher, which suggests that S100A8/A9 can also be a new marker of obesity in non-type 2 diabetes mellitus (128).

S100A8/9 is used as a marker of inflammation activity and as a predictor of the subsequent course of IBDs; in particular, fecal calprotectin testing has been applied to revolutionize IBD clinical practice with a role in differentiating IBD from functional gut disorders (129).

The level of S100A8/A9 in SF and serum is obviously higher in patients with RA than in those with OA or miscellaneous inflammatory arthritis (32). Concentration changes of S100A8/A9 in serum may be a meaningful prognostic and diagnostic biomarker for RA. Furthermore, S100A8/A9 is considered a potential marker to evaluate the responsiveness of patients with RA to biologic disease-modifying anti-rheumatic drug treatment (130).

The level of fecal S100A8/9 in infants with food allergy is twice as high as that of infants without food allergy, and S100A8/A9 may be a key contributor in promoting food allergy development in children (30). More clinical tests are still needed to prove that S100A8/9 would be a potential biological marker in hypersensitivity caused by the autoimmune system. In addition, as the serum level of S-calprotectin in patients with psoriatic arthritis increased, S100A8/9, which is associated with psoriatic arthritis pathogenesis, became a better predictor of ongoing disease than CRP or other pro-inflammatory cytokines (131).

Compared with routine inflammation indexes, including CRP, S100A8/A9 is becoming a more sensitive biomarker for inflammation activity and response to therapy (132), especially for RA, juvenile idiopathic arthritis, SLE, and a few other inflammatory diseases (8, 133, 134). The application of S100A8/9 can lead to new possibilities for diagnosis in clinical practice. Increasingly, researchers are exploring the link between S100A8/9 and other inflammatory diseases. By optimizing the detection method and practice environment, the clinical significance of S100A8/9 as a biomarker will be established.

## S100A8/A9 as a Potential Target for Treatment

S100A8 and S100A9, as well as the S100A8/A9 complex, appear to be crucial molecules during the process of inflammation, which indicates that therapies targeting these proteins may be superior to traditional ones in inflammation-associated diseases.

Tasquinimod, an oral quinoline-3-carboxamide, binds to S100A9 and the S100A8/A9 complex in the presence of Zn^2+^ and Cu^2+^ and thus blocks the interaction of S100A9 with TLR4 or RAGE, inhibiting TNF-α release in an S100A9-dependent model *in vivo* (135). Quinoline-3-carboxamide has been used with encouraging outcomes in inflammatory diseases such as type 1 diabetes (136), SLE (137), and multiple sclerosis (138).

Blockade of soluble S100A8/A9 or S100A8/A9 secretion during sepsis could represent an enlightening therapeutic strategy, as surviving patients were shown to have decreased S100A8/A9 levels compared with non-survivors (139). Targeting S100A8/A9 can also prevent liver injury as well as bacterial dissemination at an early phase during human sepsis and endotoxemia (119). However, what is different is that low-dose-S100A8-induced self-tolerance and cross-tolerance may provide a potential strategy for attenuating overwhelming pro-inflammatory cascades and enhancing antimicrobial responses during microbial sepsis (9).

Targeting S100A8/A9 relieves organ injury by decreasing tissue damage in the lung during tuberculosis (140). Similarly, targeting S100A9 could control lung inflammation and associated lung disease during IAV infection (3). In addition, in biofilm-infected recalcitrant wounds, local S100A8/A9 could be a latent pivotal molecular target in individualized adjunctive immunotherapy (141).

For RA patients, there is evidence that S100 proteins can be targeted in therapeutic approaches. S100A8 may provide an effective therapeutic strategy for reducing inflammation and preventing cartilage and bone destruction (31). Treatment with anti-S100A9 antibody improves the clinical score by 50% in RA patients (75). In murine models of arthritis, blockade of S100A8/A9 ameliorates inflammatory processes, and there is evidence that S100 proteins could also be potential targets in human arthritis patients (142).

Both of the results demonstrate that S100A8, as well as S100A9, may exert considerable influence on human hypersensitivity and could be considered a potential target. S100A9 participates in the processes of asthma by initiating and amplifying neutrophilic inflammation (143), and S100A8 plays a protective role in airway hyperresponsiveness by inhibiting airway smooth muscle contraction in asthma (144).

Furthermore, the expression of S100A8 and S100A9 in eosinophils is highly upregulated in colonic inflammation, and these proteins participate in tissue repair, which means that eosinophil-mediated effector pathways may provide new curative targets in colonic inflammation and repair, especially in IBD (145). In a previous study, we provided strong evidence that different key pathways such as NF-κB and STAT3 signaling are specifically involved in different phases, which bridge the gap between inflammation and cancer, and revealed a novel mechanism in which inflammation-induced S100A8 promoted colorectal tumorigenesis by acting upstream to activate the Akt1–Smad5–Id3 axis. We also found a protective effect of neutralizing anti-S100A9 antibody against DSS-induced colitis and AOM/DSS-induced colitis-associated cancer in a mouse model, which suggests that anti-S100A9 antibody may provide a novel therapeutic approach to treat ulcerative colitis (146–148).

In psoriasis and psoriatic arthritis, both S100A8 and S100A9 may represent good therapeutic targets (24) by regulating complement component C3 (149). However, psoriasis-like inflammatory phenotypes in the K14-Angptl6 Tg mice were not rescued by S100A9 deletion, which means decreasing S100A9 levels may not ameliorate all cases of psoriasis (150). The reason may because not all mechanisms associated with psoriasis are governed by S100A9.

S100A8 would be a good target for a new line of therapeutics against obesity-induced chronic inflammation *via* blocking the initial trigger and halting the very early events of the vicious cycle (151). In addition to targeting S100A8 and S100A9 directly, inflammation in adipose tissues is also decreased by inhibiting the TLR4 ligand and NLRP3–IL-1β signal axis (152, 153). Furthermore, S100A8 and S100A9 are able to serve as potential therapeutic targets during the inflammatory state following bariatric surgery (154).

Due to its potential involvement in atherogenesis, plaque vulnerability, ischemia-associated myocardial inflammation, and heart failure, S100A8/A9 might serve as a therapeutic target in CVD (155). S100A8/A9 can be a novel therapeutic target candidate for ruptured intracranial aneurysm (156), acute coronary syndrome (157) and so on. For example, quinoline-3-carboxamide mentioned in the preamble has been demonstrated to reduce atherosclerotic plaque size, inflammation, and vulnerability features in S100A12 transgenic hyperlipidemic ApoE^−/−^ mice (158). It is supposed that relative concentrations and posttranslational modifications of calgranulins may have distinct functional outcomes that are protective at different stages of atherogenesis in particular microenvironment (159). In addition, regulating the S1008A–SAA3–LOX-1 cascade in the disease may improve the stability of atherosclerosis and decrease clinical cardiovascular events (160).

From another aspect, in diabetes-related CVDs, targeting RAGE (161), one of the receptors of S100A8/A9, and using ABR-215757 (paquinimod) (162) both show an ability of vascular protection. As a consequence, targeting S100A8/A9 has been demonstrated to be effective, and the drugs associated with S100A8/A9 are approved for clinical testing.

In AD, upregulation of the S100A9 gene plays an important role in neuropathology and memory impairment, which can serve as a link between AD amyloid and neuroinflammatory cascades and has the potential to be a prospective therapeutic target. Thus, the reduction of S100A9 in the chronic inflammatory phase of AD may be a treatment opportunity (23, 95, 163).

It is worth mentioning that in type 1 diabetes, a chronic inflammatory disease characterized by autoimmune destruction, both S100A8 and S100A9 may be targets for therapeutics as they participate in the processes of inflammation, metabolic regulation, and autoimmunity during disease development (164).

In OA, targeting S100A8 and S100A9 could be an interesting option for future OA therapies to avoid bone loss, considering the longtime expression of these proteins in the synovium during OA (35, 165).

Since S100A8, S100A9, and the S100A8/A9 complex are all involved in the pathogenesis of most of the inflammatory diseases, it is hypothesized that targeting S100A8 and S100A9 can be used as a treatment for these diseases. Some animal experiments and clinical trials have proved this conjecture, but more evidence is needed before its widespread application in clinical practice. Therefore, there is an urgent need to understand their specific biological functions at various stages and exact molecular mechanisms in different inflammatory diseases.

## Summary

As an alarmin of inflammation, S100A8 and S100A9 are significantly increased in almost all types of inflammation. S100A8/A9 induces not only bacteriostatic but also cytokine-like effects in the local environment. Despite the substantial amounts of evidence showing the importance of S100A8/A9 in the biological functions of inflammatory disease, the defense mechanisms of calprotectin are still not very clear. With respect to functional studies, only a few detailed characterizations exist related to S100A8/A9, while there are adequate studies on S100A8 and S100A9 separately. This condition requires researchers to carry out more experiments in the future to facilitate our understanding of the S100A8/A9 heterodimer. However, purification and observation of the complex is very challenging.

Currently, S100A8/A9 has been found to play an important role in many diseases, such as inflammation, cancer, and can even be used as a typical or atypical marker to diagnose diseases or predict the progress of diseases (Table S1 in Supplementary Material). These discoveries motivate researchers to explore whether S100A8/A9 could be used as a biomarker or therapeutic target in diseases beyond inflammation and cancer.

## Author Contributions

SW, RS, ZW, ZJ, SW, and JM analyzed the literatures and studies and wrote the manuscript. The authors sincerely thank Dr. Xuemei Zhang for her support during the writing process.

## Conflict of Interest Statement

The authors declare that the research was conducted in the absence of any commercial or financial relationships that could be construed as a potential conflict of interest.
