# Supplementary material for: S100A8/A9 in Inflammation
Source: Front Immunol. 2018 Jun 11;9:1298. doi: 10.3389/fimmu.2018.01298 (PMC6004386; doi:10.3389/fimmu.2018.01298)
Supplement: Supplementary file 1 [file Table_1.PDF]

Supplementary Table 1. S100A8/A9's expression, function and utility in inflammation-related diseases

Disease	Expression	Function of S100A8/A9	Potential target for treatment	Reference
Sepsis	1. Elevated in neutrophils<br>2. Amniotic fluid (AF)	1. Promoting early bacterial spread and liver damage<br>2. Promoting endotoxin-induced shock<br>3. Inhibiting growth of related pathogens<br>4. Participating in macrophage phagocytosis and chelation by divalent cation	A diminished activation of TLR4 in non-S100A8/A9 mice during the early response to infection, resulting in less inflammation and reduced tissue injury.	(1-4)
AIDS	Lower levels in neutrophils	Inhibition of peripheral neutrophils oxidative metabolism	Mediated by adenosine metabolites.	(5)
Influenza A Virus (IAV)	Released from intact macrophages	Enhancing inflammation	1. IAV virus-stimulated (non-LPS) activated S100A9 (paracrine or autocrine) -TLR4-MyD88-IL-6, TNF-α.<br>2. Antibodies of S100A9 inhibit a pro-inflammatory response in macrophages to control of lung inflammation during IAV infection.	(6)
RA	1. Expressed in neutrophils, macrophages, PMNs, synovial fibroblasts, and chondrocytes<br>2. Elevated in the synovial fluid, especially the cartilage-pannus junctions	1. Drive severe joint inflammation and cartilage destruction<br>2. Immune modulatory<br>3. Activation of endothelial cells<br>4. Activation of leukocytes, chemotaxis, anti-microbial activity, downstream signaling with NF-κB	1. Blocking S100A8/S100A9 secretion: inhibit inflammation activity with NF-κB-activation.<br>2. S100A8/A9-TLR4: fibroblast proliferation; production of metalloproteinase (MMP)-1, IL-6, and further S100A8 and S100A9 activation of NF-kB.<br>3. S100A9-EMMPRIN: S100A9-induced monocyte/macrophage migration RAGE, Heparan sulfate, N-glycans.	(7-10)
Asthma	S100A8: elevated in lung of asthmatic rats.<br>S100A9: elevated in neutrophils.	S100A8: inhibition of airway smooth muscle (AMR) contraction to regulate Airway hyperresponsiveness (AHR).<br>S100A9: initiation and amplify the neutrophilic inflammation.	1.S100A9-induced IL-1β, IL-17 and IFN-γ: control neutrophilic inflammation to lower resistant to the corticosteroids in asthma.<br>2.S100A8-prevented Ach-induced MLC-phosphorylation: inhibits ASM-contraction	(11-13)

IBD	1. Elevated in eosinophils during colonic inflammation repair<br>2. Abundance during active IBD. 79d	1. Inhibiting the growth of various fungi and bacteria.79d<br>2. Regulating leukocyte migration, cytokine expression, and innate immune activity.s80 64-66<br>3. Healing mucosal. N17	S100A8/A9-TLR4/RAGE/surface heparan sulfate proteoglycan/carboxylated N-glycans on endothelial cells: downstream nuclear NF-κB activation 79d	(14-19)
SLE	1.Expressed in human platelets<br>2.Elevated and localized in close proximity to intracellular membranes and granules	1. Pro-inflammatory and pro-thrombotic<br>2. May be involved in vesicle trafficking and in cell communication	1. S100A8/A9-CD36: regulation of carotid artery occlusion time<br>2. S100A8/A9-RAGE: key event in development of atherosclerosis and CVD<br>3. S100A8/A9-RAGE/TLR-4: amplifying the inflammatory response<br>4. S100A9 binds to phosphatidylserine through Annexin V: membrane structures in macrophages	(20-22)
Psoriasis	Elevated in keratinocyte	1. Induction of C3 (cytokine)<br>2. Hyperproliferation of keratinocyte<br>3. Angiogeness	S100A8/A9-C3-IL-17A/IMCP-1/RANTES: lead to 9-C3-IL-1 keratinocytes and subsequently to uncontrolled immune cell activation, angiogenesis, hyperproliferation of keratinocytes	(23-27)
Obesity	Elevated in adipose cell	Induction of noresolving inflammation by secreting cytokines	S100A8/A9-TLR4-IL-1β	(28)
CVD	Elevated in endothelial	1. Cytokine and chemokine secretion<br>2. Platelet aggression<br>3. Neutrophil and monocyte recruitment<br>4. Cytokine secretion, cytoskeleton modulation, leukocyte recruitment	1. S100A8/A9-RAGE-Endothelial activation-permeability, cytokine secretion, chemokine secretion, platelet aggregation (vascular wall)<br>2. S100A8/A9-TLR4-neutrophil, monocyte recruitment and activation (vascular wall)<br>3. Hyperglycemia obesity-S100A8/A9-Mac-1 expression and affinity-neutrophilia, monocytosis (blood)<br>4. S100A8/A9-RAGE-M-CSF,GM-CSF-neutrophilia,	(20,29,30)

			monocytosis (bone marrow)	
Gout	1. Elevated in neutrophils, macrophages; synovia, tophi and sera<br>2. Correlated with progress of gout.	1. Increasing monosodium urate crystal (MSU)-induced secretion of IL-1β via TLR4<br>2. Inducing expression of IL-1β、IL-6、TNF-α through stimulating production of ROS	S100A8/A9-TLR4: inhibiting TLR4-dependend expression of cytokines.	(31,32)
Alzheimer disease	1. Elevated in activated microglia;<br>2. Accumulating in amyloid plaques	1. Activiting microglia and inducing expression of inflammatory factors, leading to upregulated β-CTF (precursor of Aβ) production via activiting BACE1/2 promoters.<br>2. Interacting with Aβ to form amyploid plaques.	1.S100A8/A9-TLR4/RAGE: inhibiting TLR4/RAGE-TNFα/IFNγ-BACE1/2-β-CTF cascade.<br>2.Blocking positive feedback as well as interaction between A8/A9 and Aβ.	(33-37)
Diabetes mellitus	Elevated in vascular endothelial, leukocytes and fibroblasts.	1. Inducing cytokine and chemokine secretion via TLR4/RAGE.<br>2. Upregulating the expression of adhesion molecules, such as Mac-1, and enhancing leukocyte-endothelial cell interaction to facilitate leukocyte migration.	S100A8/A9-TLR4/RAGE: suppressing excessive secretion of cytokines and chemokines, and reducing expression of adhesion molecules to inhibit leukocyte migration.	(30,38-40)
OA	1. Released by macrophages<br>2. Elevated in Fibroblast-like synoviocytes (FLSs)	Contribute to increasing osteophytes and help to cause joint destruction	S100A8/A9-TLR4/RAGE-cytokines-joint destruction	(9,41-44)

Supplemental Table References

1. Achouiti A, Vogl T, Urban CF, Rohm M, Hommes TJ, van Zoelen MA, et al. Myeloid-related protein-14 contributes to protective immunity in gram-negative pneumonia derived sepsis. PLoS Pathog (2012) 8(10):e1002987. doi: 10.1371/journal.ppat.1002987. PubMed PMID: 23133376; PubMed Central PMCID: PMCPMC3486918.
2. van Zoelen MA, Vogl T, Foell D, Van Veen SQ, van Till JW, Florquin S, et al. Expression and role of myeloid-related protein-14 in clinical and experimental sepsis. American Journal of Respiratory & Critical Care Medicine (2009) 180(11):1098.
3. Gao S, Yang Y, Fu Y, Guo W, Liu G. Diagnostic and prognostic value of myeloid-related protein complex 8/14 for sepsis. Am J Emerg Med (2015) 33(9):1278-82. doi:

10.1016/j.ajem.2015.06.025. PubMed PMID: 26206243.
4. Buhimschi CS, Buhimschi IA, Abdelrazeq S, Rosenberg VA, Thung SF, Zhao G, et al. Proteomic biomarkers of intra-amniotic inflammation: relationship with funisitis and early-onset sepsis in the premature neonate. Pediatric Research (2007) 61(3):318.
5. Schwartz R, Lu Y, Villines D, Sroussi HY. Effect of human immunodeficiency virus infection on S100A8/A9 inhibition of peripheral neutrophils oxidative metabolism. Biomed Pharmacother (2010) 64(8):572-5. doi: 10.1016/j.biopha.2010.03.005. PubMed PMID: 20630697; PubMed Central PMCID: PMCPMC2944411.
6. Tsai SY, Segovia JA, Chang TH, Morris IR, Berton MT, Tessier PA, et al. DAMP molecule S100A9 acts as a molecular pattern to enhance inflammation during influenza A virus infection: role of DDX21-TRIF-TLR4-MyD88 pathway. PLoS Pathog (2014) 10(1):e1003848. doi: 10.1371/journal.ppat.1003848. PubMed PMID: 24391503; PubMed Central PMCID: PMCPMC3879357.
7. Ometto F, Friso L, Astorri D, Botsios C, Raffeiner B, Punzi L, et al. Calprotectin in rheumatic diseases. Exp Biol Med (Maywood) (2017) 242(8):859-73. doi: 10.1177/1535370216681551. PubMed PMID: 27895095; PubMed Central PMCID: PMCPMC5407536.
8. Austermann J, Zenker S, Roth J. S100-alarmins: potential therapeutic targets for arthritis. Expert Opin Ther Targets (2017) 21(7):739-51. doi: 10.1080/14728222.2017.1330411. PubMed PMID: 28494625.
9. Carrión M, Juarranz Y, Martínez C, González-Álvaro I, Pablos JL, Gutiérrez-Cañas I, et al. IL-22/IL-22R1 axis and S100A8/A9 alarmins in human osteoarthritic and rheumatoid arthritis synovial fibroblasts. Rheumatology (2013) 52(12):2177-86.
10. Neumann, Elena, Lefèvre, Stephanie, Zimmermann, Birgit, et al. Rheumatoid arthritis progression mediated by activated synovial fibroblasts. Trends in Molecular Medicine (2010) 16(10):458.
11. Lee TH, Chang HS, Bae DJ, Song HJ, Kim MS, Park JS, et al. Role of S100A9 in the development of neutrophilic inflammation in asthmatics and in a murine model. Clin Immunol (2017) 183:158-66. doi: 10.1016/j.clim.2017.08.013. PubMed PMID: 28847516.
12. Xu YD, Wang Y, Yin LM, Park GH, Ulloa L, Yang YQ. S100A8 protein attenuates airway hyperresponsiveness by suppressing the contraction of airway smooth muscle. Biochem Biophys Res Commun (2017) 484(1):184-8. doi: 10.1016/j.bbrc.2017.01.033. PubMed PMID: 28088518.
13. Xu YD, Cui JM, Wang Y, Yin LM, Gao CK, Liu YY, et al. The early asthmatic response is associated with glycolysis, calcium binding and mitochondria activity as revealed by proteomic analysis in rats. Respiratory Research (2010) 11(1):107.
14. Hadar R, Itay M, Michal I, Metsada PC, Thomas V, Johannes R, et al. Transcriptome profiling of mouse colonic eosinophils reveals a key role for eosinophils in the induction of s100a8 and s100a9 in mucosal healing. Scientific Reports (2017) 7(1).
15. Boyapati RK, Rossi AG, Satsangi J, Ho GT. Gut mucosal DAMPs in IBD: from mechanisms to therapeutic implications. Mucosal Immunol (2016) 9(3):567-82. doi: 10.1038/mi.2016.14. PubMed PMID: 26931062.
16. Waddell A, Ahrens R, Tsai YT, Sherrill JD, Denson LA, Steinbrecher KA, et al. Intestinal CCL11 and eosinophilic inflammation is regulated by myeloid cell-specific RelA/p65 in mice. J Immunol (2013) 190(9):4773-85. doi: 10.4049/jimmunol.1200057. PubMed PMID: 23562811; PubMed Central PMCID: PMCPMC3969817.

17. Katsue S, Masahiro Y, Jiro Y, Kouji T, Masanori K, Hiroshi Y, et al. The S100A8/A9 heterodimer amplifies proinflammatory cytokine production by macrophages via activation of nuclear factor kappa B and p38 mitogen-activated protein kinase in rheumatoid arthritis. Arthritis Research & Therapy (2006) 8(3):R69.

18. Kerkhoff C, Eue I, Sorg C. The Regulatory Role of MRP8 (S100A8) and MRP14 (S100A9) in the Transendothelial Migration of Human Leukocytes. Pathobiology (1999) 67(5-6):230-2.

19. Kerkhoff C, Klempt M, Sorg C. Novel insights into structure and function of MRP8 (S100A8) and MRP14 (S100A9). Biochim Biophys Acta (1998) 1448(2):200-11.

20. Lood C, Tyden H, Gullstrand B, Jonsen A, Kallberg E, Morgelin M, et al. Platelet-Derived S100A8/A9 and Cardiovascular Disease in Systemic Lupus Erythematosus. Arthritis Rheumatol (2016) 68(8):1970-80. doi: 10.1002/art.39656. PubMed PMID: 26946461.

21. Esdaile JM, Abrahamowicz M, Grodzicky T, Li Y, Panaritis C, Berger RD, et al. Traditional Framingham risk factors fail to fully account for accelerated atherosclerosis in systemic lupus erythematosus. Arthritis & Rheumatology (2001) 44(10):2331-7.

22. New SE, Goettsch C, Aikawa M, Marchini JF, Shibasaki M, Yabusaki K, et al. Macrophage-Derived Matrix Vesicles: An Alternative Novel Mechanism for Microcalcification in Atherosclerotic Plaques. Circulation Research (2013) 113(1):72-7.

23. Martel BC, Litman T, Hald A, Norsgaard H, Lovato P, Dyring-Andersen B, et al. Distinct molecular signatures of mild extrinsic and intrinsic atopic dermatitis. Exp Dermatol (2016) 25(6):453-9. doi: 10.1111/exd.12967. PubMed PMID: 26841714.

24. Sakaguchi M, Murata H, Aoyama Y, Hibino T, Putranto EW, Ruma IM, et al. DNAX-activating protein 10 (DAP10) membrane adaptor associates with receptor for advanced glycation end products (RAGE) and modulates the RAGE-triggered signaling pathway in human keratinocytes. J Biol Chem (2014) 289(34):23389-402. doi: 10.1074/jbc.M114.573071. PubMed PMID: 25002577; PubMed Central PMCID: PMCPMC4156043.

25. Schonthaler HB, Guinea-Viniegra J, Wculek SK, Ruppen I, Ximenez-Embun P, Guio-Carrion A, et al. S100A8-S100A9 protein complex mediates psoriasis by regulating the expression of complement factor C3. Immunity (2013) 39(6):1171-81. doi: 10.1016/j.immuni.2013.11.011. PubMed PMID: 24332034.

26. Tanigawa H, Miyata K, Tian Z, Aoi J, Kadomatsu T, Fukushima S, et al. Upregulation of ANGPTL6 in mouse keratinocytes enhances susceptibility to psoriasis. Sci Rep (2016) 6:34690. doi: 10.1038/srep34690. PubMed PMID: 27698489; PubMed Central PMCID: PMCPMC5048131.

27. Chimenti MS, Triggianese P, Botti E, Narcisi A, Conigliaro P, Giunta A, et al. S100A8/A9 in psoriatic plaques from patients with psoriatic arthritis. Journal of International Medical Research (2016) 44(1 suppl):33-7.

28. Murray Peter J. Obesity Corrupts Myelopoiesis. Cell Metabolism (2014) 19(5):735-6. doi: 10.1016/j.cmet.2014.04.010.

29. Schiopu A, Cotoi OS. S100A8 and S100A9: DAMPs at the crossroads between innate immunity, traditional risk factors, and cardiovascular disease. Mediators Inflamm (2013) 2013:828354. doi: 10.1155/2013/828354. PubMed PMID: 24453429; PubMed Central PMCID: PMCPMC3881579.

30. Mosch J, Gleissner CA, Body S, Aikawa E. Histopathological assessment of calcification and inflammation of calcific aortic valves from patients with and without diabetes mellitus. Histology & Histopathology (2017) 32(3):293-306.

31. Holzinger D, Nippe N, Vogl T, Marketon K, Mysore V, Weinhage T, et al. Myeloid-related proteins 8 and 14 contribute to monosodium urate monohydrate crystal-induced inflammation in

gout. Arthritis Rheumatol (2014) 66(5):1327-39. doi: 10.1002/art.38369. PubMed PMID: 24470119.

32. Chiu CW, Chen HM, Wu TT, Shih YC, Huang KK, Tsai YF, et al. Differential proteomics of monosodium urate crystals-induced inflammatory response in dissected murine air pouch membranes by iTRAQ technology. Proteomics (2015) 15(19):3338-48. doi: 10.1002/pmic.201400626. PubMed PMID: 26205848.

33. Lodeiro M, Puerta E, Ismail MA, Rodriguez-Rodriguez P, Ronnback A, Codita A, et al. Aggregation of the Inflammatory S100A8 Precedes Abeta Plaque Formation in Transgenic APP Mice: Positive Feedback for S100A8 and Abeta Productions. J Gerontol A Biol Sci Med Sci (2017) 72(3):319-28. doi: 10.1093/gerona/glw073. PubMed PMID: 27131040.

34. Kummer MP, Vogl T, Axt D, Griep A, Vieira-Saecker A, Jessen F, et al. Mrp14 deficiency ameliorates amyloid beta burden by increasing microglial phagocytosis and modulation of amyloid precursor protein processing. J Neurosci (2012) 32(49):17824-9. doi: 10.1523/JNEUROSCI.1504-12.2012. PubMed PMID: 23223301.

35. Zhang C, Liu Y, Gilthorpe J, van der Maarel JR. MRP14 (S100A9) protein interacts with Alzheimer beta-amyloid peptide and induces its fibrillization. PLoS One (2012) 7(3):e32953. doi: 10.1371/journal.pone.0032953. PubMed PMID: 22457725; PubMed Central PMCID: PMCPMC3310843.

36. Wang C, Klechikov AG, Gharibyan AL, Wärmländer SKTS, Jarvet J, Zhao L, et al. The role of pro-inflammatory S100A9 in Alzheimer’s disease amyloid-neuroinflammatory cascade. Acta Neuropathologica (2014) 127(4):507-22.

37. Chang KA, Kim HJ, Suh YH. The role of S100a9 in the pathogenesis of Alzheimer's disease: the therapeutic effects of S100a9 knockdown or knockout. Neurodegener Dis (2012) 10(1-4):27-9. doi: 10.1159/000333781. PubMed PMID: 22301734.

38. Jin Y, Sharma A, Carey C, Hopkins D, Wang X, Robertson DG, et al. The Expression of Inflammatory Genes Is Upregulated in Peripheral Blood of Patients With Type 1 Diabetes. Diabetes Care (2013) 36(9):2794.

39. Bouma G, Lamtse WK, Wierengawolf AF, Drexhage HA, Versnel MA. Increased serum levels of MRP-8/14 in type 1 diabetes induce an increased expression of CD11b and an enhanced adhesion of circulating monocytes to fibronectin. Diabetes (2004) 53(8):1979-86.

40. Burkhardt K, Schwarz S, Pan C, Stelter F, Kotliar K, Von Eynatten M, et al. Myeloid-related protein 8/14 complex describes microcirculatory alterations in patients with type 2 diabetes and nephropathy. Cardiovasc Diabetol (2009) 8:10. doi: 10.1186/1475-2840-8-10. PubMed PMID: 19232095; PubMed Central PMCID: PMCPMC2654885.

41. van den Bosch MH, Blom AB, Schelbergen RF, Koenders MI, van de Loo FA, van den Berg WB, et al. Alarmin S100A9 Induces Proinflammatory and Catabolic Effects Predominantly in the M1 Macrophages of Human Osteoarthritic Synovium. J Rheumatol (2016) 43(10):1874-84. doi: 10.3899/jrheum.160270. PubMed PMID: 27481901.

42. Schelbergen RF, de Munter W, van den Bosch MH, Lafeber FP, Sloetjes A, Vogl T, et al. Alarmins S100A8/S100A9 aggravate osteophyte formation in experimental osteoarthritis and predict osteophyte progression in early human symptomatic osteoarthritis. Ann Rheum Dis (2016) 75(1):218-25. doi: 10.1136/annrheumdis-2014-205480. PubMed PMID: 25180294.

43. van Lent PL, Blom AB, Schelbergen RF, Sloetjes A, Lafeber FP, Lems WF, et al. Active involvement of alarmins S100A8 and S100A9 in the regulation of synovial activation and joint destruction during mouse and human osteoarthritis. Arthritis Rheum (2012) 64(5):1466-76. doi: 10.1002/art.34315. PubMed PMID: 22143922.

44. Liu X, Liu R, Croker BA, Lawlor KE, Smyth GK, Wicks IP. Distinctive pro-inflammatory gene signatures induced in articular chondrocytes by oncostatin M and IL-6 are regulated by Suppressor of Cytokine Signaling-3. Osteoarthritis Cartilage (2015) 23(10):1743-54. doi: 10.1016/j.joca.2015.05.011. PubMed PMID: 26045176.
